# How Much Demand for New HIV Prevention Technologies Can We Really Expect? Results from a Discrete Choice Experiment in South Africa

**DOI:** 10.1371/journal.pone.0083193

**Published:** 2013-12-30

**Authors:** Fern Terris-Prestholt, Kara Hanson, Catherine MacPhail, Peter Vickerman, Helen Rees, Charlotte Watts

**Affiliations:** 1 Department of Global Health and Development, London School of Hygiene and Tropical Medicine, London, United Kingdom; 2 Wits Reproductive Health and HIV Institute, Hillbrow, Johannesburg, Gauteng, South Africa; 3 Collaborative Research Network for Mental Health and Wellbeing in Rural and Regional Communities (CRN), University of New England, Armidale, NSW, Australia; University of Missouri-Kansas City, United States of America

## Abstract

**Background:**

For the first time in the history of HIV, new bio-medical interventions have been shown to be effective in preventing HIV transmission. For these new HIV prevention technologies (NPTs) to have an impact on the epidemic, they must be widely used. This study uses a discrete choice experiment (DCE) to: understand the relative strength of women’s preferences for product characteristics, understand the implications for substitution away from male condoms, and inform realistic modelling of their potential impact and cost-effectiveness.

**Methods:**

A DCE was conducted among 1017 women in urban South Africa. Women were presented with choices between potential women’s NPTs (microbicides, diaphragm, female condom) and ‘what I did last time’ (use or not use a condom) with different HIV and pregnancy prevention effectiveness’ and prices. Choice probabilities are estimated using the nested logit model and used to predict uptake.

**Results:**

In this high HIV prevalence setting, HIV prevention effectiveness is the main driver of uptake followed by pregnancy prevention effectiveness. For example a microbicide with poor effectiveness would have niche appeal at just 11% predicted uptake, while a highly effective microbicide (95% effective against HIV and pregnancy) would have far wider appeal (56% predicted uptake). Though women who reported not using condoms were more likely to choose the NPTs, at current very high rates of male condom use in South Africa (60%), about half of microbicide uptake is projected to be among those currently not using condoms.

**Conclusions:**

Women are very interested in NPTs, especially if highly effective in preventing HIV and pregnancy. Women in greatest need were also most likely to switch to the new products. Where products are not yet available for distribution, proxy data, such as that generated by DCEs, can bring realism to overly optimistic uptake scenarios found in many current impact models.

## Introduction

For the first time in the 30 year history of HIV, new bio-medical interventions have been shown to be effective in preventing HIV transmission [1]. These include new technologies (microbicides, also called topical pre-exposure prophylaxis) and novel applications of existing antiretroviral drugs for treatment (e.g. oral pre-exposure prophylaxis), and “treatment as prevention” to prevent new HIV infections [1]–[3]. Further down the product development pipeline are a number of multi-purpose prevention technologies which prevent multiple reproductive health indications such as HIV and pregnancy and/or sexually transmitted infections (STIs) [4], [5]. Such products are under development in the form of vaginal rings, physical barriers (e.g. cervical barriers like the SILCS diaphragm) or injectable compounds [6]. As yet, no information exists on the value women place on products providing protection against a range of outcomes, their predicted level of uptake, the impact of their introduction on the use of male condoms, or data upon which to predict use of products with multi-purpose characteristics.

In the light of these new HIV prevention technologies (NPTs), there has been much speculation and debate about the potential for ‘risk compensation’ following the introduction of a partially effective NPT [7]. This could arise where people have a ‘target risk level’ [7]; so, as a consequence of decreased risk of HIV resulting from the NPT, risky behaviour is increased, which could then undermine the effect of NPTs, and theoretically could increase an individual’s total HIV risk [7]. Lessons from the introduction of voluntary medical male circumcision and vaccine trial participation have not provided conclusive evidence on risk compensation [3], [8], [9]. To fully understand behaviour change effects, further studies will need to be undertaken during actual roll out [7]. However, in the absence of this, choice experiments eliciting hypothetical preferences for switching from existing HIV prevention product (condoms) to NPTs could provide critical insights.

To estimate the impact of the introduction of NPTs and inform decision-making on their optimal roll out strategies, mathematical models have been widely used [10]–[21]. These models tend to estimate the epidemiological impact and cost-effectiveness of these NPTs and identify the key determinants that drive these outcomes. To date such modelling studies have all assumed user uptake curves that are not dependent on product characteristics, despite numerous other studies on NPTs showing the importance of product effectiveness on user acceptability and predicted uptake [22]–[28].

This study uses a discrete choice experiment (DCE) to: explore the effect of product characteristics (HIV prevention effectiveness, pregnancy prevention effectiveness, the ability to use it in secret and price) on women’s preferences for three potential NPTs (microbicides, a cervical barrier, and the female condom) in South Africa; identify potential substitution effects; and predict the uptake of microbicides.

## Methods: The Choice Experiment

This DCE aims to identify the key attributes of NPTs, specifically microbicides, the diaphragm, and the female condom, that drive women’s willingness and ability to use them within the context of their own intimate lives. The formative research for this study took place between June and August 2005 and the survey took place in September 2005.

### Ethics Statement

This study was approved by the ethics committees of the University of the Witwatersrand in South Africa and the London School of Hygiene and Tropical Medicine, UK. Written informed consent was obtained from all participants.

### The study context

This study was conducted at the same time and in a comparable population, but in a different community, from a randomised controlled trial of microbicides, the Microbicide Development Programme 301 (MDP 301), conducted in South Africa, Zambia, Uganda, and Tanzania between 2005 and 2009 [29]. Microbicides are a class of products such as gels or tablets that a woman can insert into her vagina to reduce the risk of acquiring HIV. Although the MDP 301 trial products had no impact on HIV acquisition [30], a subsequent trial (the CAPRISA 004 trial) of a different microbicide product, found that tenofovir gel reduced HIV incidence by 39%, increasing to 54% among high adherers [3]. This was a breakthrough, with the HIV community eagerly awaiting the results of a further trial to confirm these results in 2014 [31].

Concurrent with the MDP 301 trial, the MIRA trial, which ran from 2003 to 2006, tested the effectiveness of the diaphragm in preventing HIV acquisition among women in South Africa and Zimbabwe; this trial also did not show an effect [32]. Recently the SILCS diaphragm has been developed and is expected to provide protection against pregnancy and cervical STIs similar to the traditional diaphragm [33]. It could also be used as a microbicide delivery system thus providing multi-purpose prevention. Though a new device, insights from studies of the traditional diaphragm are still likely to be informative for uptake.

At the time of this study, the price of the female condom, at 18 times that of male condoms, was considered prohibitively expensive for wide scale distribution. Female condoms were available in South Africa in a limited number of clinics [34] and were rationed by health care workers, moreover there was limited knowledge of female condoms among women in the general population. Innovations to the female condom characteristics are reducing price and there is renewed support for its distribution in South Africa [35]. It is therefore the right time to prepare for the introduction of NPTs and consider factors affecting their potential demand.

### Development of the discrete choice experiment

The DCE was developed based on an intensive qualitative phase including individual and group interviews during the three months prior to the household DCE survey. This formative phase aimed to inform the design of the DCE scenarios, and identify key product characteristics (attributes) and their variations (levels) by understanding challenges women face in accessing and using existing HIV prevention technologies and the most appropriate representation of the attributes. An example of an attribute could be price, with levels being the specific prices, say 0, 5, 10, 20. Ten focus group discussions (FGDs) were held among older (30–50 years old) and younger (18–29 years old) women, i.e. five among each. Women were recruited from clinic attendees and from within the community. The FGDs took place in the local clinic’s training room on Saturdays. Weekly FGD topic guides were finalised based on the findings from the preceding week. Subsequently, two attribute reduction workshops were held, where individual questionnaires were completed by women in a group interview setting with the aim of identifying the most important attributes of products from the long lists generated in the FGDs. Women were asked to rank lists of characteristics based on their most and least preferred options and the interpretation of alternative questionnaire wording and pictorial representations of attribute descriptions were explored to ensure the survey tool was capturing what it was intended to capture [36]. Finally, 10 individual interviews/pilot interviews were undertaken to understand women’s interpretation of the scenarios presented, such as the presentation of HIV and pregnancy prevention effectiveness. Results from the qualitative phase can be found in MacPhail et *al*. 2009 [37]. In the final design of the qualitative research, policy objectives and likely realistic levels of product attributes were considered; the final five attributes were product (microbicide, diaphragm and female condom), pregnancy prevention effectiveness, HIV prevention effectiveness, the ability to use it without the partner’s knowledge and price. The attributes and their levels can been seen in Table 1.

**Table 1:** DCE physical attributes and levels.

Alternative	A,B	C	
		Neither, I would do what I did the last time I had sex*	
Attribute	Levels		
*Product*	Microbicide (MCD), Diaphragm (DGM), Female Condom (FC)	Male Condom(MC)	No protection (None)
*Effectiveness in preventing HIV*			
(MCD, DGM)	35%, 55%, 75%, 95%	95%	0%
(FC)	75%, 95%		
*Effectiveness in preventing pregnancy*			
(MCD)	0%, 55%, 75%, 95%	95%	0%
(DGM,FC)	75%, 95%		
*Ability to use without partner’s knowledge*			
(MCD, DGM)	Cannot be used in secret, Can be used in secret	Cannot be used in secret	Can be used in secret
(FC)	Cannot be used in secret		
*Price in US dollars*			
Single use product (MCD, FC)	Free, US$0.54, US$1.04, US$2.08	Free	Free
Reuseable product (DGM)	Free, US$4.16, US$ 8.32, US$16.64	Free	Free

MCD: microbicide; DGM: Diaphragm; FC: Female condom; *Depending on what they did the last time they had sex, a card was placed over the attributes with the appropriate attribute levels for either using a male condom with its attributes, or no protection.

**HIV and pregnancy effectiveness:** The levels of expected HIV effectiveness of the product were developed based on the then-ongoing trials of single dose pre-coital microbicides (MDP301 in Uganda, Tanzania, Zambia, South Africa [30]) and the diaphragm (MIRA in South Africa and Zimbabwe [32]) and existing evidence on condom effectiveness, starting from as low as 35% reduction in HIV risk for microbicides and the diaphragm and 75% for the female condom (representing typical use and accounts for breakage and slippage) to 95% reduction in HIV risk, comparable with expected effectiveness of the male condom under perfect use. Pregnancy prevention effectiveness levels for microbicides and the diaphragm were based on discussions with the scientific teams of the MDP 301 and MIRA trials, respectively.

**Ability to use in secret:** Ability to use women’s HIV prevention technologies discreetly was reported as more important than pure covert use [38]. Pure covert use is considered where the partner cannot know of product use, whereas discreet use is defined as being able to use the product without the partner’s active participation. Just over half of women in a user trial chose to use the microbicides and diaphragm without informing their partners [39]. The female condom, though not secret, does not require the male partner’s active participation. The ability to use the product in secret was included in two levels (possible or not possible to use without partner knowing) for microbicides and the diaphragm, while male and female condom use would always be known to the partner.

**Prices:** Prices were developed based on suggestions from FGD participants for the various products and prices of condoms distributed through the public, social marketing and private sector distribution channels in South Africa (Free, US$0.54, US$1.04, and US$2.08 (South African Rand (R)0, R5, R10, R20 ))[40]. To reflect the reusability of the diaphragm and the reported higher valuation by women in the FGDs, diaphragm prices were set at four times the price levels of the other NPTs (Free, US$4.16, US$ 8.32, US$16.64 (R0, R20, R40, R80)) [40].

**Opt out:** An opt-out option was included as represented by ‘I would do what I did last time I had sex’, which was then represented by what women had reported earlier in the questionnaire (having used or not having used a condom during their last sex-act). The attribute levels of the opt-out were then presented consistent with the attributes of the reported protection in last sex-act (Table 1, alternative C).

The final design of the choice sets was a compromise between a behavioural model of choice, emphasising realism of choice sets and alternatives, and statistical models, emphasising the need for a parsimonious design. This was obtained using the ORTHOPLAN procedure in SPSS.

### The choice task

**Figure 1:**
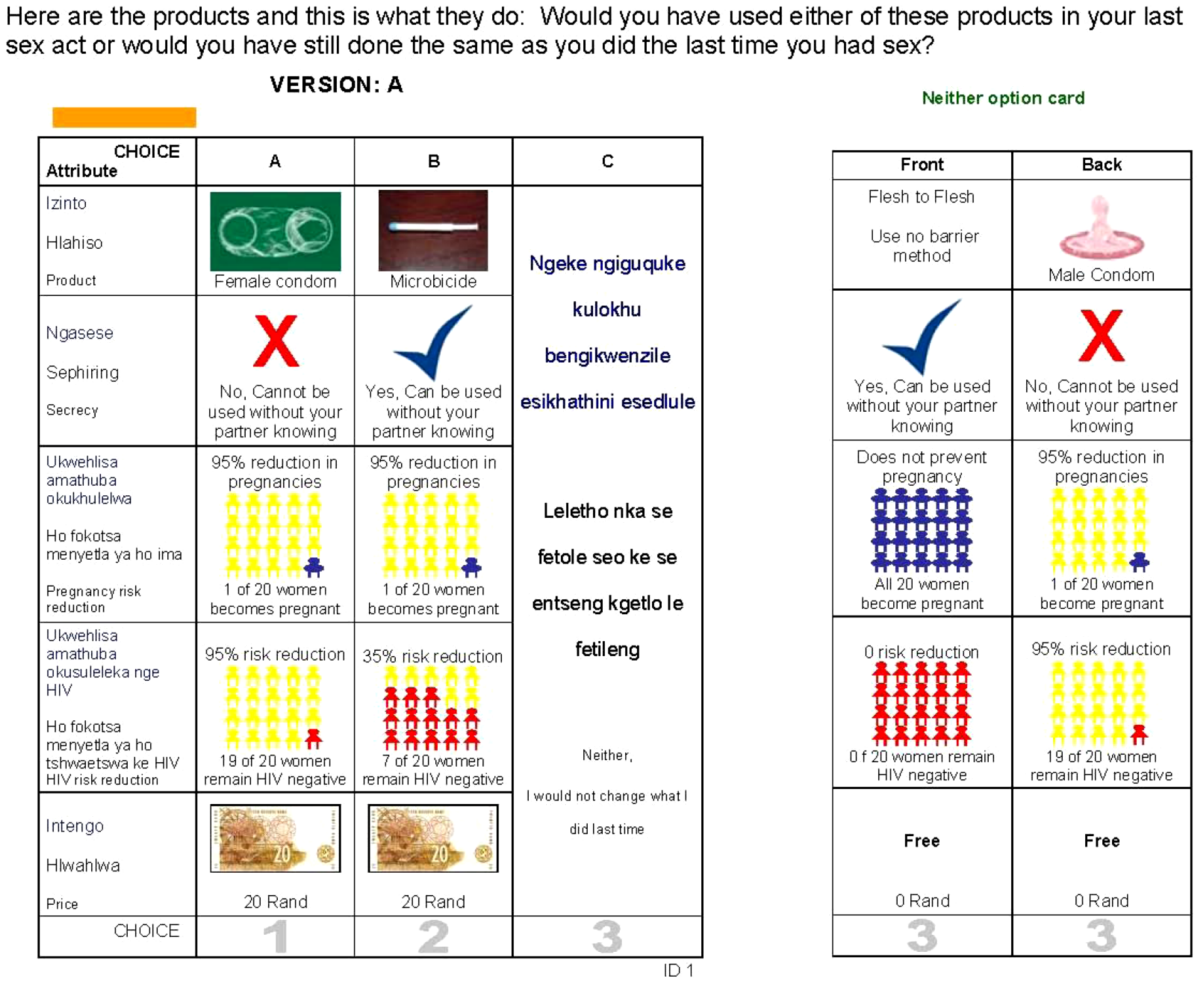
An example of the choice task.

The unlabelled DCE consisted of six choice tasks with three alternatives in the format presented in Figure 1. This DCE asked women to think back to their last sex-act, and choose if they would have switched to one of the new options (A or B) or if they would have done the same that they actually did in their last sex-act (alternative C) (Figure S1 in File S1).

A two-sided opt-out card presented attribute levels for condoms, or non use of condom. The card was placed in the utmost right column as the opt-out choice. The interviewer could thus place the card over the third option, with the side up to correspond to the scenario of their last -act (having used a condom or not), and specify the appropriate attribute levels. The availability of female condoms was very low, so by framing the opt-out as male condom or use no HIV protection and the new products as the main alternatives, the full range of technologies for HIV prevention that were available or in trials in 2005 were represented.

### Sampling and data collection procedures

Women were eligible to participate if they were between 18 and 45 years of age and had been sexually active in the past six months. Sample randomisation occurred at three levels: geographical area, household, and within households, if there was more than one eligible woman in a selected household. A representative sample of 1017 eligible women from three adjacent communities in Ekurhuleni Metropolitan Municipality south-east Johannesburg were interviewed in their homes. Of 2026 households approached, 29% did not have any eligible women, 10% were not at home after three visits. Refusal rate was just 3%, though an additional 3% postponed and could also be considered refusals. A further 4% had missing reasons, other reasons or were incorrectly sampled. The sample realisation (number of completed interviews per household approached) was 51%.

This paper-based survey was administered by 17 locally recruited and trained female fieldworkers. The community survey questionnaire consisted of four sections. The first collected background data on the respondent, including indicators of socio-economic status (education level, type of housing, and an asset index). The second section collected reproductive histories, including experience with existing HIV prevention technologies: what they use, where they obtained it, if they had or had not used a condom in their last sex-act. This describes the ‘neither’ option. The reproductive histories also included information about contraceptives, life stage questions (current type(s) of sexual partnership(s) and desire for pregnancy), and the respondent’s perceived HIV risk. The method that was used last time reveals preferences for (and ability to use) condoms - the existing method. The third section presented an introduction to the range of HIV prevention technologies and elicited preferences for products and their attributes. A male condom, female condom, a diaphragm, and a microbicide applicator filled with lubricant were available for respondents to inspect, touch and ask questions about. This was provided to reduce the hypothetical nature of the products. An additional DCE was also administered to collect women’s preferences for distribution outlet characteristics and promotional messaging, results from this are reported elsewhere [41]. The last section consisted of questions evaluating the interview and eliciting participant feedback on the questionnaire.

Self-reported behaviour data can be subject to social desirability bias and choice experiments subject to hypothetical bias, potentially leading to upwardly biased results. Hypothetical bias refers to the inconsistency between reported behaviour and observed behaviours, when the reported choice does not require real action [9]. In the case of NPTs another layer of hypothetical bias is introduced by the fact that the products do not exist on the market and have not been experienced by the participants. This study aimed to reduce hypothetical bias in two ways. Firstly, by framing the scenarios within the context of the respondent’s last sex-act (what Henscher calls “referencing around a real world experience” [9]), the researchers aimed to provide a less hypothetical use scenario. Secondly, respondents were also shown actual product prototypes and were able to handle them, but could of course not experience them in a real setting within the context of this study. Social desirability bias relates to respondents’ desire to report socially acceptable behaviour and underreport higher risk behaviours, and has been widely observed in the field of HIV research on self reported sexual behaviours [8]. Efforts were made to emphasise to respondents that there were no right or wrong answers. However, in urban South Africa people are inundated on a daily basis with safer sex messaging. It is likely that this has both biased upwards reports of condom use in the last sexact, and women’s choices in favour of alternatives with very high HIV effectiveness. However, we must also not underestimate the prominence of HIV in South African women’s daily lives and the value women place on having *usable* HIV prevention options. Due to these potential biases, this study’s uptake predictions may represent the upper limits of what may be expected in reality. This can then generate an upper bound uptake prediction for use by impact modellers.

The questionnaire was translated into the two most common non-English languages in the recruitment areas (Sesotho and isiZulu); then translated back for validation.

## Methods: Statistical Analysis

### Sample generalisability

A review of the household and individual characteristics of the study participants was undertaken using basic descriptive statistics. Generalisability was assessed by comparing women’s socio-demographic characteristics with those from two nationally representative surveys in South Africa: the South Africa Demographic and Health Survey from 2003 [42] and the South African National HIV Prevalence, HIV Incidence, Behavioural and Communication Survey, 2005 (known as the Nelson Mandela Survey) [43].

### Participant feedback

In addition to open ended questions on participants’ experience of the survey, we asked three closed ended questions: What did you think of the scenarios we presented? Easy, difficult, confusing; Were they interesting or boring? Were there too many or too few? We present summary statistics on these to provide an overview of participant feedback.

### Model specification

The basic workhorse for estimating discrete choice models remains the multinomial logit (MNL) model. However, it is based on a rather stringent restriction on substitution behaviour between alternatives, known as “independence of irrelevant alternatives” (IIA) (for more detail see Green 2000 p. 864 or Henscher et al p. 518) [44], [45]. The nested logit (NL) model is a popular alternative to the MNL model as it relaxes the IIA assumption while maintaining ease of computation. It depicts choices in hierarchical levels with partitioned choice sets. Within a partition (branch), IIA must hold, but between the branches the model relaxes the IIA assumption. The NL can explicitly model ‘non-participation’ (the no-change choice) [46], which is particularly attractive to this application. Further details of this model and the NL tree structure can be found in File S1.

The outputs of discrete choice models are relative utilities. The simplest form of the model incorporates only design attributes in the utility function, i.e. main effects: product (microbicide (*MCD*), diaphragm (*DGM*), female condom (*FEMC*)); ability to use in secret (*SECR);* effectiveness against HIV (*HIV*); effectiveness against pregnancy (*PREG);* and *Price* (specified as ln(price+1)) (*PRICE*) and interactions between product and effectiveness (DGMPREG, MCDPREG, DGMHIV, MCDHIV). The price specification followed extensive exploration, using partworths to allow fully free utilities for each price and identifying the functional form of price that fit best. We settled on the logarithm of price+1. The 1 was added as log of price 0 cannot be obtained. Non-linear relationships for pregnancy and HIV effectiveness were explored but not identified. Interactions between attributes were explored, in particular whether women value pregnancy and HIV prevention effectiveness differently between the products.

The utility function of main effects for switching, depending on if the not switch option was a condom or nothing, is represented by:



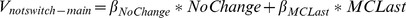
.

The main effects utility function for the NPTs is represented by:



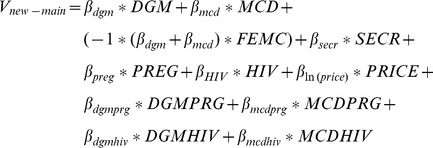
.

Beyond main effects we expect a number of variables related to women’s situations and experiences to be important in their willingness and ability to use NPTs. In unlabelled (generic) designs, variations among women in preferences for the products and their attributes are explored by including interactions with women’s socio-demographic characteristics. Note that in labelled designs, interactions can be entered directly into the utility function. It is hypothesised that the choice to switch to any of the NPTs could be affected by the utility of their current practice (use of a male condom in last sex-act (*MCLastS*)), having ever experienced difficulties getting a partner to use a male condom (*DiffMC*), or cohabiting (*Cohab*). This creates the following utility function for the choice to switch or not:



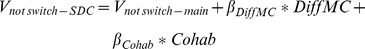
.

It is hypothesised that the preference for:

- products is different for women who used a condom relative to those who did not (DGM*MCLastS, MDC*MCLastS, FEMC*MCLastS );
- being able to use a product in secret is different for women who had experienced difficulties getting their partner to use a male condom in the past (SECR*DiffMC);
- pregnancy prevention effectiveness is different for women who are living with their partners (PRG*Cohabiting) than those who are not, because of their life stage/circumstances relating to the consequence (positive or negative) of conception;
- HIV prevention effectiveness is different for women who consider themselves at higher risk of becoming HIV infected (HIV*self perceived risk of HIV);
- HIV prevention effectiveness is different for women who are cohabiting (HIV*Cohab).

This generates the following utility function for the choice between NPT alternatives: 
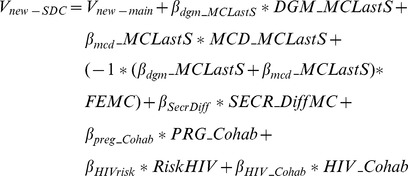



Effects coding has been used for categorical variables. This means that the coefficients must be interpreted as divergence from the imposed mean of 0 for that attribute. To retrieve the coefficient of the omitted category, we take [–1*∑coefficients other levels in attributes]. Not including a constant in the estimation imposes a mean utility of 0 [47], with coefficient values representing relative strength of preferences. A constant is used for labelled experiments to capture the mean value of the label, however for generic experiments this is meaningless since A and B have no useful meaning, and would capture a preference for the left hand side option over the right hand side option.

NLOGIT 3.0 was used for estimation. Using its simulation command, predicted market uptake for microbicides with varying characteristics is estimated for condom and non-condom users, and a weighted average is used to predict population uptake, based on the women’s reported condom use at last sex-act (60.4%) in 2008 [48].

## Results

### Data description

The sample included 1017 adult women (18 to 45 years old), with an average age of 31.5 years (Table 2). The largest category of participants had entered but not completed secondary school (45%); only 27% finished secondary school. More than a third were employed and just under a third lived in a household that had a working car. This is close to the national averages among adult urban women in South Africa.

**Table 2:** Sample descriptive statistics.

	This sample*	Demographic & Health Survey 2003 [42]
		Urban sample
**Socio economic status indicators**		
Age (age)	31.5 years	
Education: started but did not complete secondary school	45%	47%
Employed part or full time (emplyd)	35%	42%
Ownership of a car	31%	33%
**Reproductive health profiles**		
Number of children	1.8	1.6
Ever use of contraceptives	72%	73%
Current use of modern contraception	40%	51%
Condom used at last sex-act (MCLastS)	31%	29%
Female condom – ever seen	39%	
Female condom – ever used	3%	
Cohabiting with sexual partner (Cohab)	55%	
Ever experienced difficulties getting partner to use condoms (DiffMC)	38%	
Self-perceived risk of HIV **– high (RiskH)	22%	
Self-perceived risk of HIV – medium (RiskM)	26%	
Self-perceived risk of HIV – low (RiskL)	23%	
Self-perceived risk of HIV – no (RiskNo)	26%	

The denominator of the proportions for ‘This Sample’ are based on the full sample, ranging from 1012 to 1017 to account for some missing responses.** The risk question was posed as: “Do you consider yourself at high medium, low, or no risk of getting HIV or becoming re-infected?” No further quantification was provided and therefore was the individuals subjective valuation of these categories. Risk is an ordinal variable that is used as a cardinal measure in the model estimates, valued as 0 for ‘No risk’ to 3 for ‘High risk’.

The sexual and reproductive lives of women in this sample were also comparable to other representative surveys [42] [43], with 1.8 children per woman, and 72% having ever used modern contraception and 31% reporting having used a condom in their last sex-act. Though 39% had ever seen a female condom, less than 3% had ever used one. Current use of contraception appears lower than the average urban sample, at 40% versus 51% [42]. Just over half of the sample was cohabiting with their partner and 38% had ever experienced difficulties getting their partner to use a condom. There was a fairly even distribution across the various categories of self perceived HIV risk.

Participants were asked about their preferred HIV prevention product, after receiving a detailed description. Microbicides were chosen as preferred product by 48% of respondents, followed by the diaphragm chosen by 28% of respondents. The female and male condoms were least preferred, chosen by 13% and 10%, respectively. Though these results give relative preferences for the full product, DCE can separate out drivers of these choices based on specific product characteristics.

Table 3 provides an overview of the choices made according to women’s characteristics that are hypothesised to have an effect on their choice behaviour. ‘Change’ represents choosing one of the alternatives of NPTs, while ‘not change’ represents the alternative ‘do what I did last time I had sex’. Of the 6101 choices (6 choice sets*1017 women and 1 missing response) possible across the full sample of respondents, if left to chance, choices would fall equally across the three alternatives: two-thirds of the responses would be expected for ‘change’ and one-third would be expected for ‘not change’. Seventy-four percent of choices were in favour of a NPT, 26% were for ‘not change’. Women who used a male condom appear less likely to change to one of the NPTs: in 56% of their choices they chose one of the NPTs versus 83% of women who did not use protection. This suggests that preferences for the NPTs are likely to differ between women who were and were not able to use a male condom in their last sex-act. Other groups of women with high switching responses were: those currently using contraceptives (80%), those who experienced difficulties using condoms (82%), and those who perceive themselves at high risk of HIV (83%).

**Table 3:** Switching responses by women's characteristics.

		Change		Not-change	
		Count	%	Count	%
All		4,539	74%	1562	26%
Condom used at last sex-act (MCLastS)	No	3,466	83%	727	17%
	Yes	1,073	56%	835	44%
Cohabiting with sexual partner (Cohab)	No	1,926	70%	810	30%
	Yes	2,613	78%	746	22%
Ever experienced difficulties getting partner to use condoms (DiffMC)	No	2,624	69%	1156	31%
	Yes	1,890	82%	401	18%
Employed (emplyd)	No	2,902	73%	1058	27%
	Yes	1,637	76%	504	24%
Self-perceived risk of HIV (RiskH)	High	1,105	83%	232	17%
(RiskM)	Medium	1,198	77%	362	23%
(RiskL)	Low	1,043	74%	361	26%
(RiskNo)	None	1,019	65%	547	35%
		Means		Means	
Household SES (SEShh)		–0.006		0.018	
Age (age)		31.86		30.58	
Years of education (EducYrs)*		10.00		10.14	

The original variable was ordinal in terms of level of education attended and completed. This was transformed into an average number of years in education to obtain a continuous variable.

### Potential reporting bias and participant feedback

Though the interview was intended to be undertaken in private, participants sometimes encouraged the interview to go ahead despite the presence of others (16% of interviews, mostly in the presence of children). As the vast majority were done in private, we do not expect a large impact on the results.

To allow for review of reporting bias, in 20% of the sample a DCE-like scenario was included with the three potential current alternatives: male condom, female condom or use no barrier method, with their respective attribute levels. Of women who reported using a male condom in their last sex-act, 59% reported they would have used a male condom and 33% chose the female condom; however of those who did not use a condom only 13% chose the no condom option. This suggests that women’s choices were based more on their intentions for a future sex-act than on what would have happened in their last sex-act.

At the end of the DCE survey, participants were asked about their experience of making the DCE choices. About three-quarters found them easy (74%), 23% found them confusing, and 3% found them difficult. The choices were considered interesting by 95%, boring by less than one percent and neither by almost five percent. However 42% stated there were too many scenario choices.

### Estimation results

**Preferences:** Table 4 shows the results of the MNL and NL models without (models I and II, respectively) and with socio-demographic characteristics interactions (models III and IV, respectively). The likelihood ration (LR) test was used to identify the best fitting model, i.e. reject a restricted model. The basic MNL model (model i) is rejected in favour of the more flexible NL model (model 2). Subsequently model 2 is rejected in favour of model IV, which allowed for preferences to vary by women’s characteristics. From here onwards we focus on model IV.

**Table 4:** Estimation of determinants of preferences for new HIV prevention technologies and their attributes with interactions.

	Model I			Model II			Model III			Model IV				
	Multinomial Logit			Nested Logit			Multinomial Logit			Nested Logit				
	Coeff.		Std. err	Coeff.		Std. err	Coeff.		Std. err	Coeff.			Std. err	
*Main effects*														
Diaphragm (DGM)	0.590	**	0.235	0.619	**	0.293	0.597	**	0.242	0.636	**		0.289	
Microbicide (MCD)	0.364	*	0.218	0.741	***	0.280	0.284		0.225	0.604	**		0.276	
Female condom (FEMC)†	–0.953	***	0.368	–1.360	***	0.472	–0.882	**	0.379	–1.240	***		0.464	
Ability to use in secret (SECR)	0.040		0.026	0.047		0.032	0.062	**	0.027	0.068	**		0.032	
Pregnancy effectiveness (PRG)	1.431	***	0.159	1.757	***	0.195	1.457	***	0.163	1.741	***		0.195	
HIV effectiveness (HIV)	3.285	***	0.159	4.439	***	0.214	2.974	***	0.171	3.697	***		0.230	
LN(Price) (LNP)	–0.093	***	0.015	–0.133	***	0.019	–0.088	***	0.016	–0.121	***		0.019	
*Attribute interactions*														
DGM*PRG	–0.195		0.223	–0.081		0.281	–0.163		0.229	–0.076			0.277	
MCD*PRG	–0.346	**	0.169	–0.502	**	0.208	–0.315	*	0.173	–0.449	**		0.206	
FEMC*PRG†	0.541	*	0.286	0.583		0.361	0.478		0.293	0.526			0.355	
DGM*HIV	–0.543	***	0.186	–0.616	***	0.223	–0.521	***	0.191	–0.569	***		0.221	
MCD*HIV	0.261		0.183	–0.012		0.230	0.312	*	0.188	0.077			0.227	
FEMC*HIV†	0.282		0.280	0.628	*	0.354	0.209		0.288	0.492			0.348	
*Interactions with women’s characteristics*														
DGM*Used condom last sex-act (MCLastS)							0.133	***	0.035	0.145	***		0.042	
MCD*MCLastS							–0.053	*	0.031	–0.048			0.038	
FEMC*MCLastS†							–0.080	**	0.035	–0.097	**		0.042	
SECR*ever difficulties negotiating condoms (DiffMC)							0.092	***	0.023	0.101	***		0.027	
PRG* cohabiting							–0.087		0.080	–0.141			0.094	
HIV* self perceived risk of HIV							0.290	***	0.037	0.494	***		0.074	
HIV*Cohab							–0.176		0.109	–0.351	**		0.137	
LNP*employed (EMP)							0.038	***	0.011	0.044	***		0.016	
Choose ‘do the same as last time’ (C) (NOSWITCH)	2.948	***	0.203	0.548	**	0.225	3.063	***	0.211	1.092	***		0.219	
C*MCLastS							0.632	***	0.036	0.614	***		0.035	
C*DiffMC							–0.267	***	0.036	–0.250	***		0.035	
C*Cohab							–0.177	*	0.102	–0.142	**		0.062	
Inclusive values –Change				0.315	***	0.043				0.416	***		0.042	
Not Change				1.000		.....(Fixed				1.000			.....(Fixed	
N			6,101						6,065					
Ll-function	–5,907.479			–5,821.433			–5,547.393			–5,484.865				
			Model 1 versus 2							Model 2 versus 4				
LR df			1							11				
LR test stat			172.093							673.14				
LR p-value			0.00							0.00				

is significant at a P-value<0.1. ** is significant at a P-value<0.05. *** is significant at a P-value<0.01.

retrieved coefficient by swapping the omitted category.

**Figure 2:**
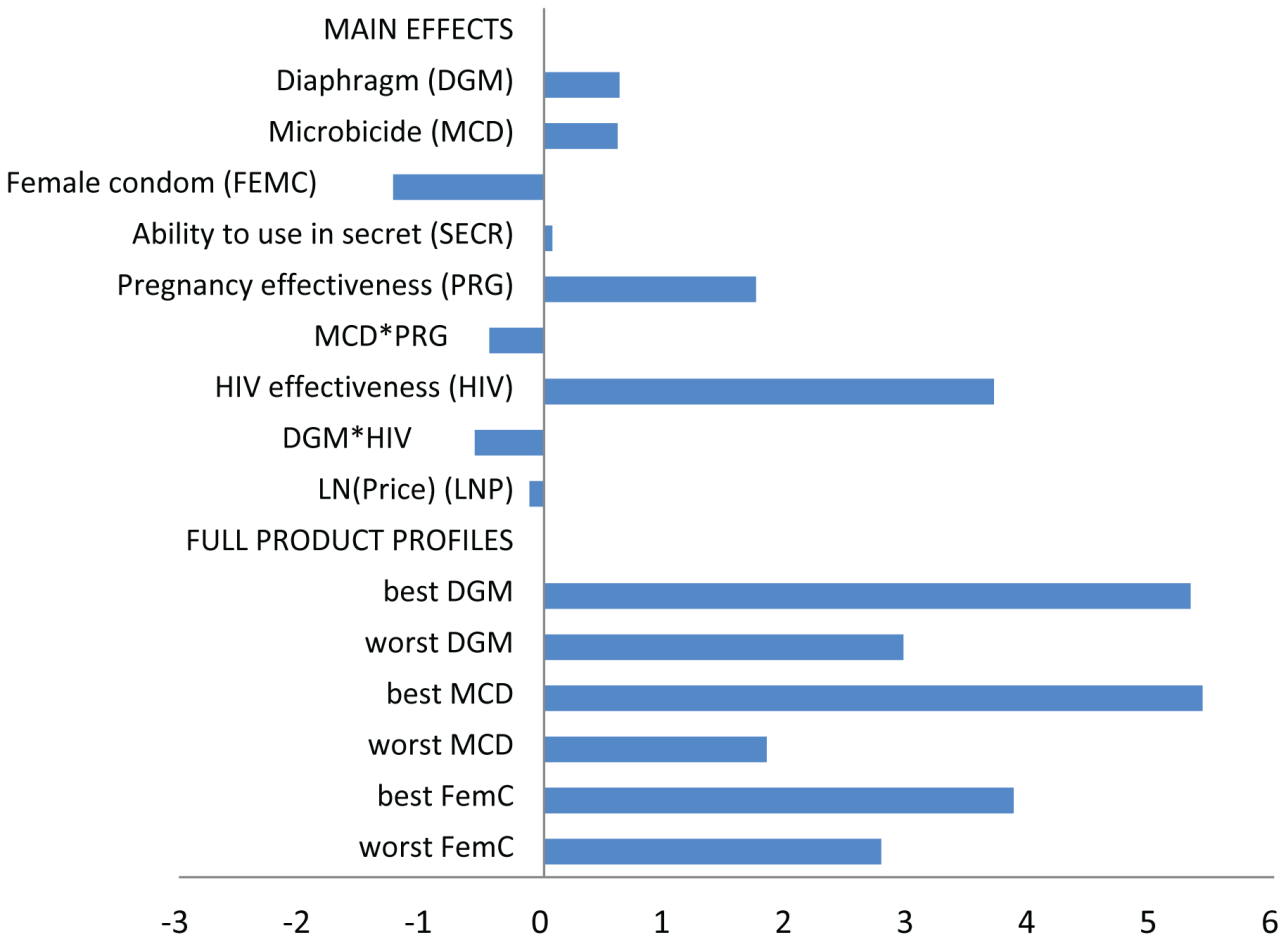
Relative preferences for products and their characteristics. Note: All full product preferences are based on free distribution. Best profiles are specified as microbicides and the diaphragm able to be used in secret and with 95% protection against pregnancy and HIV for all products. Worst profiles are specified as not being able to be used in secret and the lowest product specific effectiveness included in the experiment (i.e. prevention effectiveness: 75% for the diaphragm and female condom and 0% for microbicides; for HIV prevention effectiveness: 35% for the diaphragm and microbicide and 75% for the female condom).

The utilities from Table 4 are presented graphically in Figure 2. The top section of Figure 2 shows the relative strength of preferences for products and their attributes based on model IV, while the bottom section shows the highest and lowest valuations for each of the three products based on their possible attributes (secrecy and prevention effectiveness’). It is worth noting that by using effects coding the average utility for all attributes is set to 0.

Of the different products, the preferences for the diaphragm and microbicides were very positive and of similar magnitude, while the female condom was significantly less preferred. This pattern varied from the directly elicited preferences for products, where microbicides were chosen as preferred product by 48% of respondents, followed by the diaphragm chosen by 28% of respondents. The female and male condoms were least preferred, chosen by 13% and 10%, respectively. DCE can separate out drivers of these choices based on product characteristics.

Secrecy has a significantly positive, but small, effect on choice probability, while price was significantly negative, consistent with economic theory. The most important characteristics were the level of protection against pregnancy and HIV. The negative interaction terms between diaphragm and HIV and between pregnancy and microbicides, respectively, shows that women find the HIV effectiveness of the diaphragm less important and the pregnancy effectiveness of microbicides less important. Synergies between preferences for pregnancy- and HIV- effectiveness were explored by including their interaction terms in an earlier model, but these terms were small in magnitude and did not approach statistical significance and therefore not included in the final model specification (results available upon request).

The relative utilities of the best and worst product packages were estimated, see figure notes for how these were defined. Although the diaphragm and microbicides have similar best product valuations, the worst diaphragm and female condom are preferred to the worst microbicide due to the restrictions on their lowest pregnancy effectiveness levels (75%) thus placing a floor on their relative utilities. Microbicide preferences were elicited including product profiles that provided no protection against pregnancy.

Table 4 presents the full model results which allow for exploration if one characteristic can compensate for another. The main effects show clear trade-offs that women would be willing to make. For example, a non-contraceptive microbicide would need to have 82% HIV effectiveness to be equal to a 55% effective diaphragm with 75% pregnancy protection.

A number of interactions are included to explore the effect of women’s characteristics on their product valuations (Table 4). How women’s values for these products are affected by their current HIV prevention options/choices was explored by interacting the NPT with women’s reported condom use in their last sex-act. Having used a condom in the last sex-act implies using a condom is a feasible option, however having not used one may mean that it is not an option or that it is an option but the choice was not to use one. Women who used a condom last time have higher marginal valuations for the diaphragm and lower valuations for the female condom than women who did not use a condom. Secrecy was significantly more important to women who had ever experienced difficulties getting a partner to use a condom.

Women who were living with their partners had less strong preferences (lower marginal utilities) for HIV prevention effectiveness, potentially reflecting that cohabiting women were not as worried about HIV. Their preferences for pregnancy prevention, however, were not significantly lower than non-cohabiting women. Women who considered themselves at higher risk of acquiring HIV valued HIV prevention effectiveness more strongly. And lastly employed women had less negative coefficients for price, which implies higher willingness to pay, consistent with economic theory.

The choice to switch from what a woman did last time she had sex represents either newly protected sex-acts, if the woman had not used a condom in her last sex-act, or it represents substitution away from the condom, if she had used a condom. The value of not switching is allowed to differ if the last sex-act was or was not protected by a condom. If the woman used a condom, her relative utility is *C+C_MCLastS*, and if she did not it is *C-C_MCLastS*. Generally, the high value of the coefficient shown for not switching suggests the expected reporting bias towards switching may not be as strong as hypothesised. Women who used a condom value not switching three and a half time times more than those who did not use a condom. Having had difficulties in negotiating condoms (*DiffMC*), living with a partner (*Cohab*), and currently using contraceptives (*Contr*) decrease the relative utility of not-changing and thus also its probability. A woman with these characteristics and who did not use a condom in their last sex-act is predicted to have a near 0 utility for the *Not Switch* alternative (1.092-.614--0.25--0.142 = 0.08). In contrast, a not-cohabiting woman who has never had a problem using condoms, used a condom in her last sex-act and is not using contraception has a relative utility of 2.10 for not switching, thus is far less likely to take up one of the NPTs.

**Figure 3:**
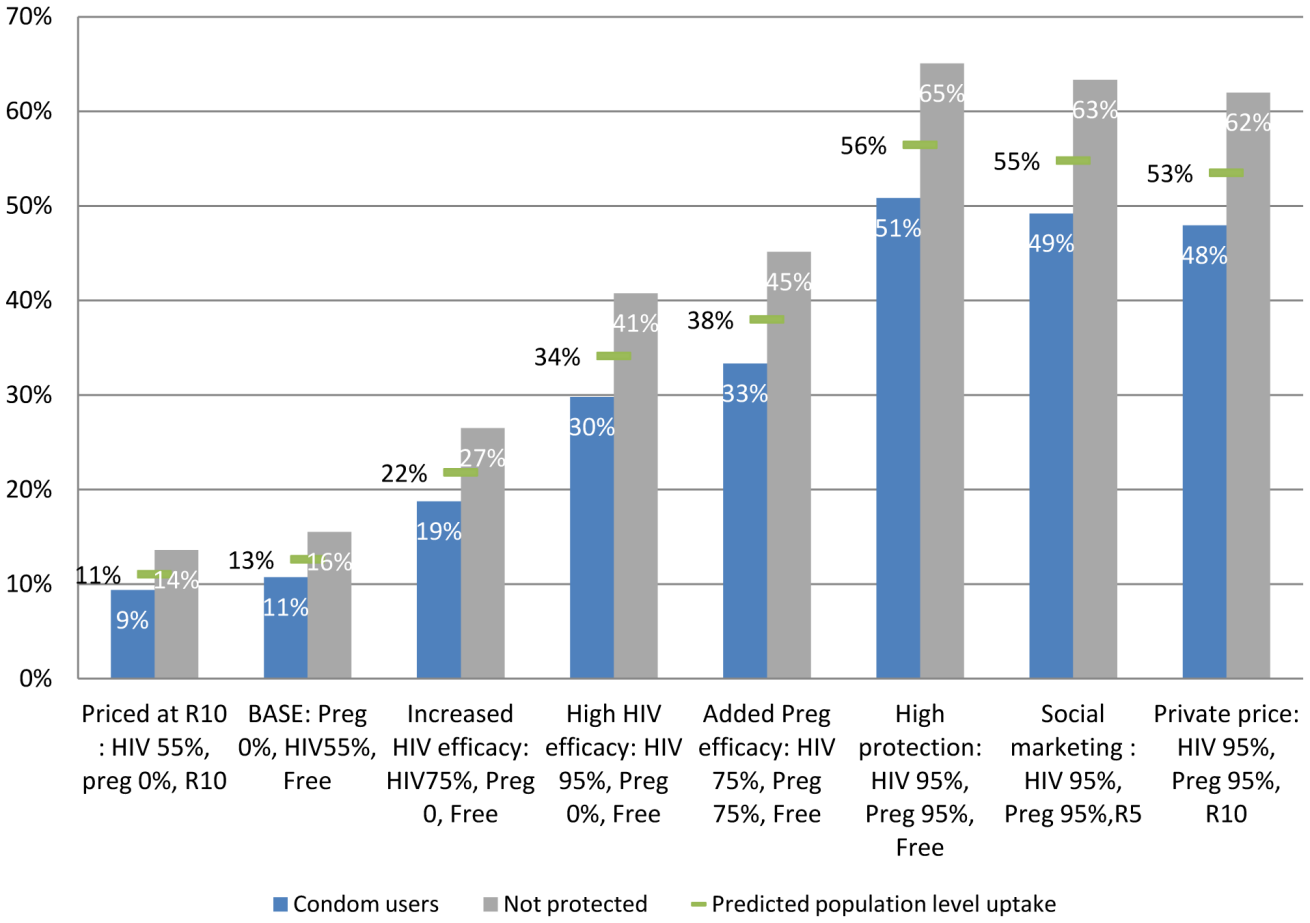
Predicted uptake of microbicides among women who had and had not used a condom. Figure 3 shows the probability of switching to a microbicide with different levels of HIV prevention effectiveness, pregnancy prevention effectiveness and price, according to whether she had used a condom or not in her last sex-act and predicted population level uptake. The choice is between the microbicide and a free female condom (with 95% protection against HIV and pregnancy), or neither. The light bars on the left are the probabilities for women who reported having used a condom in their last sex-act, on the darker bars on the right are the probabilities for women who had not used a condom in their last sex-act, the vertical line shows the predicted population level. The base case is modelled close to the CAPRISA 004 trial effectiveness results (54% effective against HIV and no pregnancy effectiveness). On the very left, it can be seen that an expensive microbicide with low prevention effectiveness would have a low probability of being chosen. As the product characteristics improve (towards the right of the figure), women find the product becomes more and more attractive relative to the female condom or what they did last time.

**Substitution and predicted uptake:** Figure 3 presents predicted uptake of a microbicide with varying characteristics. From the choice probabilities, it can be seen that for all levels of the product attributes, women who had not used a male condom considered microbicides a more attractive choice than women who had used condoms. The HIV prevention effectiveness of a product played an important role in women’s choices, with a low projected uptake of a microbicide with poor effectiveness (11%), increasing to 34% for a product with high effectiveness against HIV and no effectiveness in preventing pregnancy. Pregnancy prevention was also important to women and would enhance the attractiveness of a microbicide, facilitating its use to a very high level: up to 65% among those not using a condom. This shows that the effectiveness of microbicides in both HIV and pregnancy prevention will be a major determinant of their uptake by women, driving uptake levels from 9% to 51% if a woman had used a condom or from 14% to 65% for those who had not used a condom in their last sex-act.

## Discussion

This paper has focussed on identifying how women value different physical product characteristics for technologies to prevent HIV infection and predicting uptake levels. It is the first study to go beyond qualitative acceptability studies to quantify women’s preferences for NPTs and their trade-offs between product characteristics. The nested logit model applied uses a two-step approach in which the choice to use a NPT (substitution from what they did last time) is modelled separately from the choice among the NPTs, which allows us to explore potential substitution away from male condoms, a concern raised by policy makers and potential users alike [7], [49].

When choosing among the NPTs, microbicides and the diaphragm are generally preferred to the female condom. Although ability to use the product in secret may have a very small impact on women’s choices in general, this characteristic is important to women who had experienced difficulties getting men to use a condom. Women who had used a condom appeared more satisfied with their status quo and were less likely to try alternative methods, while women who had ever experienced difficulties getting their partner to use a condom and/or were living with their partners were more likely to try the NPTs.

HIV prevention effectiveness is the characteristic most important to women and appears to dominate most choices. Women were able to understand the relative importance of the range of effectiveness levels presented, indicated by low predicted uptake of products with low effectiveness, consistent with Gafos *et al.* [49]. Although HIV effectiveness is more than twice as important as pregnancy prevention, it is especially important to women who consider themselves at higher risk of becoming HIV infected. This is consistent with a number of other stated preference studies on preference for new HIV prevention interventions [23]–[28].

Researchers in the field of NPT acceptability have raised the issue of the large divergence between reported acceptability and subsequent use [50]. In this study, though 90% of women expressed interest in trying a microbicide, overall uptake in urban South Africa is projected to be far more modest, suggesting around one in eight women would be willing to use a 55% effective gel (about the effectiveness of tenofovir 1% gel in the CAPRISA 004 trial [3] ) with no pregnancy protection; this is likely to reach a niche market segment. A highly effective microbicide (95% protection against HIV) is likely to be far more attractive, potentially providing protection for about 40% of women who were not using condoms. A product with both high HIV protection and high pregnancy protection (95% each) may have wide interest, potentially providing prevention for around half of urban South African women, with highest uptake among those not using condoms (up to 65%). As mentioned earlier, these estimates should be considered upper bounds, given the potential for upward biased reporting of women’s likelihood of using new products.

**Figure 4:**
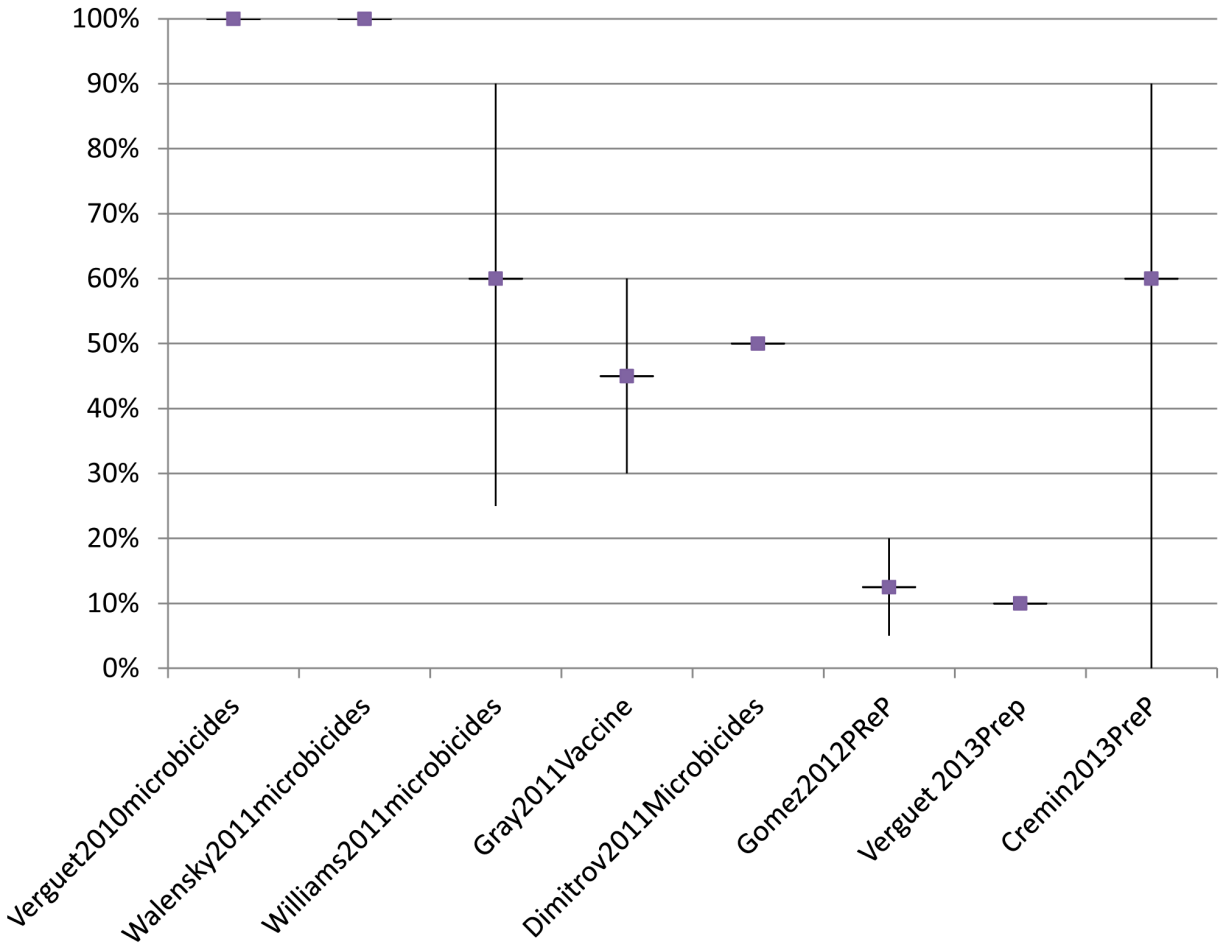
Overview of coverage assumptions in modelling impact and cost-effectiveness oral prep, microbicides and vaccines (2010 –**2013).** The middle box represents the main estimate, with the vertical lines showing ranges included.

Figure 4 shows the assumptions recent (2010–2013) modelling studies have made around uptake of oral pre-exposure prophylaxis, microbicides and vaccines coverage [10]–[13], [15], [17]–[19]. Early studies aiming to model the potential epidemiological impact assumed particularly high values for uptake and/or use. Though we see more realistic coverage levels being modelled over time, none of the existing studies take into account the interaction between product effectiveness and uptake.

This study suffers from a number of limitations. Reporting biases are inherent in survey based data, and may be of greater concern when collecting intimate sexual data. Though all efforts were made to undertake the interviews in private, 16% of interviews were undertaken in the present of others. The impact of the presence of others on choice responses will be explored in future analysis of these data. This study use an experimental design which was the state of the art in 2005, however many advances have been made with DCE experimental design approaches since then. If repeated today, we would use the NGENE package to generate an efficient design using pilot data to generate parameter priors. This would have generated a far smaller design and have allowed for a smaller sample size [51].

Great advances been made in HIV prevention and treatment in South Africa since these data were collected in 2005. We have seen a dramatic increase in reported condom use and the expansion of ART for treatment far beyond expectations, which may change valuations. The changes in context could mean HIV prevention technologies are more acceptable, potentially leading to higher uptake, or could reduce the relative value of microbicides. Pre-exposure prophylaxis and treatment as prevention have been shown to be highly effective in preventing HIV among those who have high adherence [3]. These approaches were not part of this DCE, as such is it not possible to predict uptake for the full HIV prevention arsenal. With the addition of pre-exposure prophylaxis and treatment as prevention, one would expect uptake of all options to be lower, while providing higher overall coverage. However, the NPTs that are being developed, such as vaccines or long acting injectable microbicides could completely alter the landscape of HIV prevention.

It is well known that product attributes and individual characteristics provide only partial insights into product acceptability. It is thus important to interpret the uptake predictions as *potential* uptake, depending on levels of access, distribution strategies employed and critical other household, partner, contextual and organisation factors, as conceptualised by Mensch et al. [50]. Factors relating to women’s preferences for distribution outlets and product promotion have also been analysed within the context of a sister study [52] and can provide some further insights into access and uptake, but the full range of use factors needs to be taken into account when considering potential introduction strategies. Moreover, we have learned from recent HIV prevention trials, such as MTN 003 [53], that willingness to try a new product or be enrolled in a trial is very different from high adherence/ consistent use at every sex-act, which is so critical to product effectiveness and epidemiological impact.

This study has shown how much women value pregnancy prevention, thus calling for continued investment in finding products women can use to protect themselves both from HIV *and* pregnancy, as noted by Holt [54]. Providing such multi-indication prevention is likely to provide additional motivation for high levels of adherence among women. We have shown how product effectiveness drives uptake and should therefore be explicitly accounted for when modelling the impact of NPTs. This study could provide provisional parameter values for women, which could be improved with new DCE data providing estimates of uptake for the full range of NPTs by their characteristics.

## Conclusions

This study estimates the importance of product characteristics on women’s likelihood of using a range of NPTs. It showed that above all, women want highly effective products to protect themselves from HIV. Women’s demand for such a product would be greatly increased if NPTs could also prevent pregnancy. Though most women expressed interest in these NPTs, it is women who were not using condoms that were most keen to try them. Although even a partially effective product is likely to provide important protection for some women, it is critical to not overestimate the uptake of a partially effective product. This emphasises the importance of explicitly modelling the likely interaction between product effectiveness and levels of predicted uptake when modelling the epidemiological impact of introducing any of the NPTs currently being considered for wide scale introduction and using DCEs to collect preference data on the full suite of NPTs now available.

## Supporting Information

File S1 **File contains supporting information files, including Figure S1: The nested logit tree structure.**
(DOC)

## References

[1] Baeten JM, Donnell D, Ndase P, Mugo NR, Campbell JD, et al (2012) Antiretroviral prophylaxis for HIV prevention in heterosexual men and women. N Engl J Med 367: 399–410. 22784037 10.1056/NEJMoa1108524 PMC3770474

[2] Cohen M, Chen Y, McCauley M, Gamble T, Hosseinipour M, et al (2011) Prevention of HIV-1 infection with early antiretroviral therapy. N Engl J Med 365: 493–505. 21767103 10.1056/NEJMoa1105243 PMC3200068

[3] Karim QA, Karim SS, Frohlich JA, Grobler AC, Baxter C, et al. (2010) Effectiveness and Safety of Tenofovir Gel, an Antiretroviral Microbicide, for the Prevention of HIV Infection in Women. Science Epub: http://www.sciencemag.org/cgi/content/abstract/science.1193748v1193741. 10.1126/science.1193748 PMC3001187 20643915

[4] Friend DR, Doncel GF (2010) Combining prevention of HIV-1, other sexually transmitted infections and unintended pregnancies: development of dual-protection technologies. Antiviral research 88: S47–S54. 21109068 10.1016/j.antiviral.2010.09.005

[5] Thurman AR, Clark MR, Gustavo F Doncel (2011) Multipurpose Prevention Technologies: Biomedical Tools to Prevent HIV-1, HSV-2, and Unintended Pregnancies. Infect Dis Obstet Gynecol 2011. 10.1155/2011/429403 PMC3152961 21836811

[6] Harrison PF, Hemmerling A, Romano J, Whaley KJ, Young Holt B (2013) Developing multipurpose reproductive health technologies: an integrated strategy. AIDS Res Treat 2013: 790154. 23533733 10.1155/2013/790154 PMC3600264

[7] Underhill K (2013) Study designs for identifying risk compensation behavior among users of biomedical HIV prevention technologies: Balancing methodological rigor and research ethics. Soc Sci Med. 10.1016/j.socscimed.2013.03.020 PMC4047426 23597916

[8] Phillips AE, Gomez GB, Boily M-C, Garnett GP (2010) A systematic review and meta-analysis of quantitative interviewing tools to investigate self-reported HIV and STI associated behaviours in low- and middle-income countries. Int J Epidemiol 39: 1541–1555. 20630991 10.1093/ije/dyq114

[9] Henscher D (2010) Hypothetical bias, choice experiments and willingness to pay. Transport Res Part B-Meth 44: 735–752.

[10] Williams BG, Karim SSA, Karim QA, Gouws E (2011) Epidemiological impact of tenofovir gel on the HIV epidemic in South Africa. J Acquir Immune Defic Syndr 58: 207–210. 21654503 10.1097/QAI.0b013e3182253c19 PMC3175282

[11] Walensky RP, Park JE, Wood R, Freedberg KA, Scott CA, et al (2012) The cost-effectiveness of pre-exposure prophylaxis for HIV infection in South African women. Clin Infect Dis 54: 1504–1513. 22474224 10.1093/cid/cis225 PMC3334365

[12] Verguet S, Walsh JA (2010) Vaginal microbicides save money: a model of cost-effectiveness in South Africa and the USA. Sex Transm Infect 86: 212–216. 20522634 10.1136/sti.2009.037176

[13] Verguet S, Stalcup M, Walsh JA (2013) Where to deploy pre-exposure prophylaxis (PrEP) in sub-Saharan Africa? Sexually Transmitted Infections. 10.1136/sextrans-2012-050891 23912819

[14] Hallett TB, Baeten JM, Heffron R, Barnabas R, de Bruyn G, et al (2011) Optimal uses of antiretrovirals for prevention in HIV-1 serodiscordant heterosexual couples in South Africa: a modelling study. PLoS Med 8: e1001123. 22110407 10.1371/journal.pmed.1001123 PMC3217021

[15] Gray RT, Ghaus MH, Hoare A, Wilson DP (2011) Expected epidemiological impact of the introduction of a partially effective HIV vaccine among men who have sex with men in Australia. Vaccine 29: 6125–6129. 21703320 10.1016/j.vaccine.2011.06.061

[16] Granich R, Kahn JG, Bennett R, Holmes CB, Garg N, et al (2012) Expanding ART for treatment and prevention of HIV in South Africa: estimated cost and cost-effectiveness 2011–2050. PLoS One 7: e30216. 22348000 10.1371/journal.pone.0030216 PMC3278413

[17] Gomez GB, Borquez A, Caceres CF, Segura ER, Grant RM, et al (2012) The potential impact of pre-exposure prophylaxis for HIV prevention among men who have sex with men and transwomen in Lima, Peru: a mathematical modelling study. PLoS Med 9: e1001323. 23055836 10.1371/journal.pmed.1001323 PMC3467261

[18] Dimitrov DT, Boily MC, Baggaley RF, Masse B (2011) Modeling the gender-specific impact of vaginal microbicides on HIV transmission. Journal of Theoretical Biology 288: 9–20. 21840324 10.1016/j.jtbi.2011.08.001 PMC3184649

[19] Cremin I, Alsallaq R, Dybul M, Piot P, Garnett G, et al (2013) The new role of antiretrovirals in combination HIV prevention: a mathematical modelling analysis. AIDS 27: 447–458. 23296196 10.1097/QAD.0b013e32835ca2dd

[20] Bärnighausen T, Bloom DE, Humair S (2012) Economics of antiretroviral treatment vs. circumcision for HIV prevention. Proceedings of the National Academy of Sciences of the United States of America 109: 21271–21276. 23223563 10.1073/pnas.1209017110 PMC3535659

[21] Alsallaq RA, Baeten JM, Celum CL, Hughes JP, Abu-Raddad LJ, et al (2013) Understanding the potential impact of a combination HIV prevention intervention in a hyper-endemic community. PLoS One 8: e54575. 23372738 10.1371/journal.pone.0054575 PMC3553021

[22] Cameron M (2013) The marginal willingness-to-pay for attributes of a hypothetical HIV vaccine. Vaccine 31: 3712–3717. 23747452 10.1016/j.vaccine.2013.05.089

[23] Galea JT, Kinsler JJ, Salazar X, Lee SJ, Giron M, et al (2011) Acceptability of pre-exposure prophylaxis as an HIV prevention strategy: Barriers and facilitators to pre-exposure prophylaxis uptake among at-risk peruvian populations. Int J STD AIDS 22: 256–262. 21571973 10.1258/ijsa.2009.009255 PMC3096991

[24] Kinsler JJ, Cunningham WE, Nureña CR, Nadjat-Haiem C, Grinsztejn B, et al (2012) Using conjoint analysis to measure the acceptability of rectal microbicides among men who have sex with men in four south american cities. AIDS Behav 16: 1436–1447. 21959986 10.1007/s10461-011-0045-5

[25] Lee SJ, Brooks RA, Newman PA, Seiden D, Sangthong R, et al (2008) HIV vaccine acceptability among immigrant Thai residents in Los Angeles: A mixed-method approach. AIDS Care 20: 1161–1168. 18608068 10.1080/09540120701855375 PMC2828810

[26] Lee SJ, Newman PA, Comulada WS, Cunningham WE, Duan N (2012) Use of conjoint analysis to assess HIV vaccine acceptability: Feasibility of an innovation in the assessment of consumer health-care preferences. Int J STD AIDS 23: 235–241. 22581945 10.1258/ijsa.2011.011189 PMC3372064

[27] Newman PA, Lee SJ, Duan N, Rudy E, Nakazono TK, et al (2009) Preventive hiv vaccine acceptability and behavioral risk compensation among a random sample of high-risk adults in los angeles (La voices): Research briefs. Health Serv Res 44: 2167–2179. 19780857 10.1111/j.1475-6773.2009.01039.x PMC2796320

[28] Newman PA, Roungprakhon S, Tepjan S, Yim S (2010) Preventive HIV vaccine acceptability and behavioral risk compensation among high-risk men who have sex with men and transgenders in Thailand. Vaccine 28: 958–964. 19925897 10.1016/j.vaccine.2009.10.142

[29] Nunn A, McCormack S, Crook A, Pool R, Rutterford C, et al (2009) Microbicides Development Programme: design of a phase III trial to measure the efficacy of the vaginal microbicide PRO 2000/5 for HIV prevention. Trials 10: 1–12. 19860888 10.1186/1745-6215-10-99 PMC2774685

[30] McCormack S, Ramjee G, Kamali A, Rees H, Crook AM, et al (2010) PRO2000 vaginal gel for prevention of HIV-1 infection (Microbicides Development Programme 301): a phase 3, randomised, double-blind, parallel-group trial. Lancet 376: 1329–1337. 20851460 10.1016/S0140-6736(10)61086-0 PMC2956883

[31] FACTS consortium (2012) FACTS 001 Study.

[32] Padian NS, van der Straten A, Ramjee G, Chipato T, de Bruyn G, et al (2007) Diaphragm and lubricant gel for prevention of HIV acquisition in southern African women: a randomised controlled trial. Lancet 370: 251–261. 17631387 10.1016/S0140-6736(07)60950-7 PMC2442038

[33] Schwartz JL, Ballagh SA, Creinin MD, Rountree RW, Kilbourne-Brook M, et al (2008) SILCS diaphragm: postcoital testing of a new single-size contraceptive device. Contraception 78: 237–244. 18692615 10.1016/j.contraception.2008.04.118

[34] Beksinska ME, Smit JA, Mantell JE (2012) Progress and challenges to male and female condom use in South Africa. Sex Health 9: 51–58. 22348633 10.1071/SH11011 PMC3286127

[35] Holt K, Blanchard K, Chipato T, Nhemachena T, Blum M, et al.. (2013) A nationally representative survey of healthcare provider counselling and provision of the female condom in South Africa and Zimbabwe. BMJ Open 3. 10.1136/bmjopen-2012-002208 PMC3612751 23512836

[36] Terris-Prestholt F (2010) Determinants of women's uptake of new barrier methods for HIV prevention in urban South Africa: London School of Hygiene and Tropical Medicine (University of London).

[37] MacPhail C, Terris-Prestholt F, Kumaranayake L, Ngoako P, Watts C, et al (2009) Managing men: women's dilemmas about overt and covert use of barrier methods for HIV prevention. Cult Health Sex 11: 485–497. 19479490 10.1080/13691050902803537

[38] Montgomery CM, Lees S, Stadler J, Morar NS, Ssali A, et al (2008) The role of partnership dynamics in determining the acceptability of condoms and microbicides. AIDS Care 20: 733–740. 18576176 10.1080/09540120701693974

[39] Luchters S, Chersich MF, Jao I, Schroth A, Chidagaya S, et al (2007) Acceptability of the diaphragm in Mombasa Kenya: a 6-month prospective study. Eur J Contracept Reprod Health Care 12: 345–353. 17853168 10.1080/13625180701516989

[40] OANDA (2013) OANDA currency converter.

[41] Terris-Prestholt F, Kumaranayake L, MacPhail C, Rees H, Watts C (2009) Variation in women’s preferences for microbicide distribution strategies in South Africa: Analysis of a discrete choice experiment. IHEA. Beijing.

[42] Department of Health, Medical Research Council, OrcMacro (2007) South Africa Demographic and Health Survey 2003. Pretoria: Department of Health Republic of South Africa.

[43] Shisana O, Simbaya L (2002) Nelson Mandela/HSRC study of HIV/AIDS. Cape Town: Human Sciences Research Council.

[44] Greene W (2000) Econometric Analysis. Upper Saddle River, New Jersey: Prentice-Hill.

[45] Henscher D, Rose J, Greene W (2005) Applied Choice Analysis: A Primer. Cambridge: Cambridge University Press.

[46] Brau R, Bruni ML (2008) Eliciting the demand for long-term care coverage: a discrete choice modelling analysis. Health Economics 17: 411–433. 17763359 10.1002/hec.1271

[47] Cohen J, Cohen P, West S, Aiken L (2003) Applied Multiple Regression/ Correlation Analysis for the Behavioural Sciences. London Lawrence Erlbaum Associates, Publishers.

[48] Shisana O, Rehle T, Simbayi L, Zuma K, Jooste S, et al.. (2009) South African National HIV Prevalence, Incidence, Behaviour and Communication Survey, 2008 A Turning Tide Among Teenagers? Cape Town: HSRC Press.

[49] Gafos M, Mzimela M, Ndlovu H, McCormack S, McGrath N (2012) How effective is effective enough? Opinions of potential end-users of microbicides from rural South African community.. AIDS care 10.1080/09540121.2012.722604. 10.1080/09540121.2012.722604 PMC3630452 22971053

[50] Mensch B, Van der Straten A, Katzen L (2012) Acceptability in microbicide and PrEP trials:current status and reconceptualization. Curr Opin HIV AIDS 7: 534–541. 23032737 10.1097/COH.0b013e3283590632 PMC4026162

[51] ChoiceMetrics Pty Ltd (2010) Ngene 1.1.

[52] Terris-Prestholt F, Kumaranayake L, MacPhail C, Rees H, Watts C (2008) Facilitating microbicide use in South Africa: using women’s preferences to inform distribution strategies, Abs 247. Microbicides 2008. New Delhi, India.

[53] Marrazzo J, Ramjee G, Nair G, Palanee T, Mkhize B, et al. Pre-exposure Prophylaxis for HIV in Women: Daily Oral Tenofovir, Oral Tenofovir/Emtricitabine, or Vaginal Tenofovir Gel in the VOICE Study (MTN 003); 2013 March 3-6; Atlanta, Georgia, USA.

[54] Holt BY, Kilbourne-Brook M, Stone A, Harrison P, Shields WC (2010) Multipurpose prevention technologies for sexual and reproductive health: gaining momentum and promise. Contraception 81: 177–180. 20159171 10.1016/j.contraception.2009.11.009

