# Supplementary material for: How Much Demand for New HIV Prevention Technologies Can We Really Expect? Results from a Discrete Choice Experiment in South Africa
Source: PLoS One. 2013 Dec 30;8(12):e83193. doi: 10.1371/journal.pone.0083193 (PMC3875434; doi:10.1371/journal.pone.0083193)
Supplement: File S1 — File contains supporting information files, including Figure S1: The nested logit tree structure. (DOC) [file pone.0083193.s001.doc]

**Appendix 1: The nested Logit Model**

If we start from the utility function for a choice alternative, *i*, based on random utility theory, where utility, *U*, is made up of a systematic component, *V*, and a random component, ε, representing unobserved differences in tastes: $U_{i} =V_{i} +\varepsilon_{i}$. *V* is the sum of individual *q*’s objective utilities, *ß,* of the service/benefit set of attributes of *i* with attributes *k* and the subjective utilities, *X,* of consuming *ik*: $\text{V}_{\text{iq}}=\sum_{k=1}^{K} \beta_{ik}X_{ikq}$.

The NL model allows the variance of $\varepsilon_{i}$to differ between the branches (nests), but not within. This means that $\varepsilon_{i}$is the sum of the unobservable utility at the branch level $\varepsilon_{j}$and at the elemental alternatives level $\varepsilon_{k\left|j\right.}$. The utilities are then modelled separately at the different levels and can be partitioned into two choices: 1. Participate, Not participate, with an associated utility $U_{j}$; and 2. If participate, the choice is between the NPT alternative, and their associated utilities $U_{k\left|j\right.}$.

$\varepsilon(j,k)=\varepsilon_{j}+\varepsilon_{k\left|j\right.}$ , $U(j,k)=U_{j}+U_{k\left|j\right.}$ and $V(j,k)=V_{j}+V_{k\left|j\right.}$

The unconditional probability of choosing a specific alternative $U_{j,k}$is the sum of the marginal probability at the branch level $P_{j}$and the conditional probability at the elemental alternative level, $P_{k\left|j\right.}$[[1]](#footnote-2).

$$P(j,k)=P_{j}+P_{k\left|j\right.}$$

The scale parameter is the part of the variance of the unobserved utility that is allowed to vary between nests (for more detail, see or ), and provides the link between these levels. The inclusive value is the ratio of the scale parameters of the upper $\lambda_{j}$ to the lower level$\mu_{k}$.

$$IV=\frac{\lambda_{j}}{\mu_{k}}$$

For identification purposes, one of the scale parameters must be normalised to 1, this is usually done at the upper (branch) level. The IV provides the basis for testing the appropriateness of the NL model. The closer it is to 1, the closer the levels are to having the same variance in their error terms: if it is equal to 1, the model collapses to a MNL model. The closer it is to 0 the greater the perceived similarities between the alternatives within the nest.


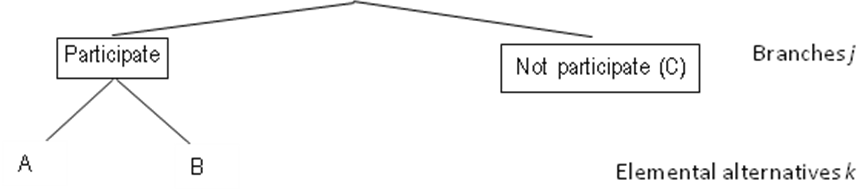


Figure S1: The nested logit tree structure

1. Henscher D, Rose J, Greene W (2005) Applied Choice Analysis: A Primer. Cambridge: Cambridge University Press.

2. Brau R, Bruni ML (2008) Eliciting the demand for long-term care coverage: a discrete choice modelling analysis. Health Econ 17: 411-433.

1. Calculation of probabilities from utility parameters is not as straightforward in the NL model. As they entail extensive equations it has been chosen not to present them or calculate them. Instead probabilities are obtained using the *Prob* command in NLOGIT. The predicted shares are also obtained from NLOGIT’s simulation command. [↑](#footnote-ref-2)
